# Laparoscopic treatment of an abdominoscrotal hydrocele: A case report

**DOI:** 10.1016/j.ijscr.2021.106668

**Published:** 2021-12-16

**Authors:** Toshifumi Hosoda, Shigeki Ishioka, Kohei Hijikata

**Affiliations:** Department of Surgery, Teikyo University Hospital, 2-11-1, Kaga, Itabashi-ku, Tokyo 173-8606, Japan

**Keywords:** ASH, Abdominoscrotal hydrocele, Abdominoscrotal hydrocele, Laparoscope, Children

## Abstract

**Introduction:**

Abdominoscrotal hydrocele (ASH), a composite of scrotal and abdominal hydroceles connected through the inguinal canal, is rare and no consensus regarding its mechanisms and surgical treatments has been reached to date.

**Presentation of the case:**

We report a case of an 11-month-old boy with a large ASH. Ultrasonography and magnetic resonance imaging (MRI) revealed a huge hydrocele (maximum length: 8 cm). The patient underwent laparoscopic percutaneous extraperitoneal closure (LPEC) and the orifice of the processus vaginalis (PV) was completely closed. The postoperative course was uneventful. Follow-up ultrasonography and MRI in the first postoperative year showed no recurrence of ASH.

**Discussion:**

An ASH with a length >8 cm is considered rare in pediatric patients. There is no consensus regarding its etiology and surgical intervention is selected according to the patient's condition and the characteristics of ASH. We opted to perform early surgical intervention considering the ASH size and the adverse effects on testicular development. LPEC helped identify the condition and location of the ASH and allowed safe and reliable operation of the large intrapelvic hydrocele. In patients with no PV patency, a change in approach from LPEC to an open anterior approach should be considered even if LPEC is feasible.

**Conclusion:**

This case provides valuable insight into successful LPEC of a large ASH without any complications, highlighting the importance of elucidating the morphological mechanisms and making an accurate diagnosis and the challenges associated with these processes.

## Highlights

• A huge abdominoscrotal hydrocele (ASH) is a rare condition in a child.
• ASH frequently exhibited a firm adhesion to the processus vaginalis.
• Laparoscopic percutaneous extraperitoneal closure does not require the detachment.
• Laparoscopic percutaneous extraperitoneal closure is a helpful and safe technique.

## 1 Introduction

Abdominoscrotal hydrocele (ASH), a composite of scrotal and abdominal hydroceles connected through the inguinal canal, is rare and accounts for approximately 0.4–3.1% of all hydroceles [1]. The first case of ASH was reported by Dupuytren et al. in 1834 [2] and more than 150 years have passed since the first case of pediatric ASH was reported by Syme et al. in 1861 [3]. Nevertheless, no consensus regarding its mechanisms and surgical treatments has been reached to date. Importantly, a recent study has highlighted the morphological mechanisms of ASH and the efficacy of surgical procedures using laparoscopy, which can be evaluated safely and in detail [4]. We describe a novel case involving successful surgical treatment of a large ASH in a child using laparoscopic percutaneous extraperitoneal closure (LPEC) without any complications.

## 2 Case report

**Fig. 1:**
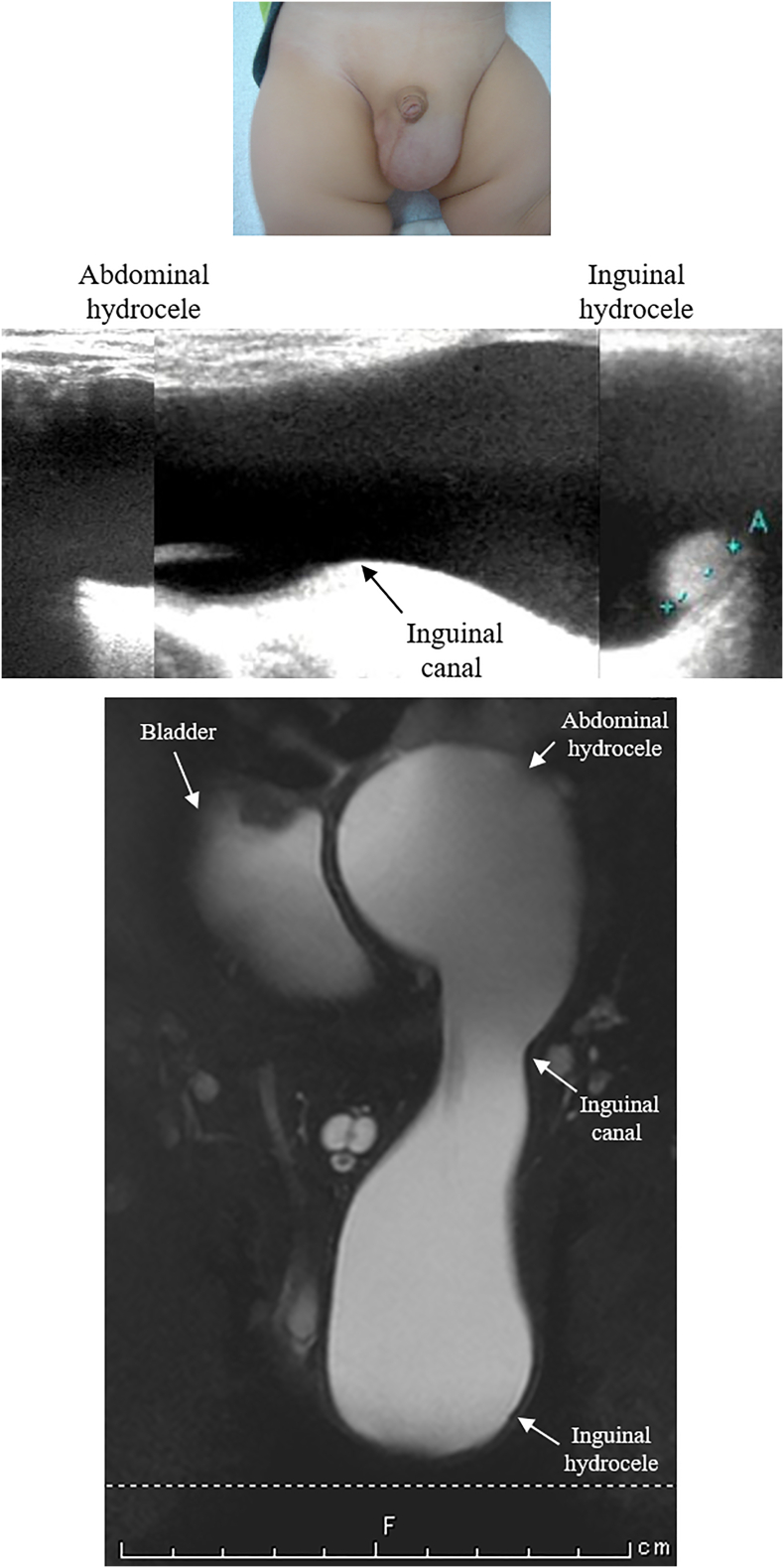
Abdominoscrotal hydrocele (ASH). (A) Development of ASH. (B) Ultrasonography findings. (C) Magnetic resonance imaging findings.

An 11-month-old boy presented with left inguinoscrotal swelling that had begun since birth. He had no medical history. During the initial physical examination, there was a translucent mass in the left scrotum (Fig. 1A). Ultrasonography showed a huge hydrocele (8 cm) extending from the swollen scrotum to the abdominal cavity via the internal inguinal canal and a compressed testicle at the bottom of the scrotum (Fig. 1B). Magnetic resonance imaging (MRI) revealed high signal intensity of a unilocular hydrocele on T2-weighted images (Fig. 1C). Additionally, the abdominal hydrocele was compressing the bladder to the right (Fig. 1C). We opted to perform LPEC, as we reasoned that the ASH exhibited a firm adhesion of the spermatic cord to the processus vaginalis (PV) and LPEC could allow performing a high ligation with minimum detachment.

**Fig. 2:**
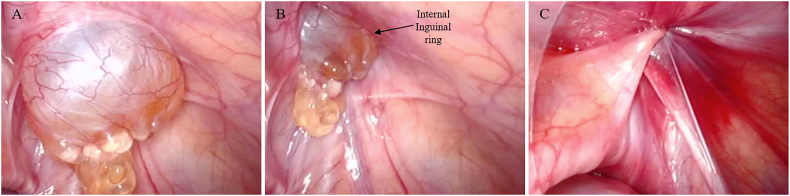
Intraoperative findings of laparoscopic percutaneous extraperitoneal closure (LPEC). (A, B) A whitish mass into the abdominal cavity through the left internal inguinal ring. (C) Successful complete closure of processus vaginalis by LPEC.

LPEC was performed as described previously [5], [6]. The procedure was performed under general anesthesia. A paraumbilical block was added for postoperative pain relief. After a small skin incision at the umbilicus, an expandable 5-mm trocar was inserted. The abdominal cavity was filled with CO2 at a flow rate of 0.8–1.0 L/min to a level of 6–7 mm Hg. A 30° telescope was inserted through the umbilical port. After obtaining adequate view of the abdominal cavity, the assistant's working forceps was inserted via the lateral abdominal wall. The operative bed was tilted to the head-down (10–15°) to relocate the intestine from the internal inguinal regions to the upper abdomen. Subsequently, abdominal inspection of the internal inguinal regions was performed to detect the PV and ASH. We confirmed the “Springback sign” (SBB sign) wherein the hydrocele swelled into the abdominal cavity when the scrotum was compressed and shrank when the scrotum was released. Laparoscopic findings showed a whitish mass in the abdominal cavity through the left internal inguinal ring (Fig. 2A and B). A long and straight 19-gauge needle (Lapaherclosure; Hakko Medical Co., Tokyo, Japan) that has a wire loop to hold a nonabsorbable suture thread at the tip of the needle was inserted via the surface of the inguinal region. After performing the extraperitoneal purse suture around the internal inguinal ring without the puncture of ASH and pulling out both the ends of the suture thread, the PV orifice was completely closed (Fig. 2C). After removing the forceps, the umbilical wound was closed. The postoperative course was uneventful. No recurrence of ASH was found in the first postoperative year.

## 3 Discussion

Although childhood ASH was first reported in 1861 [3], its etiology is still controversial. The following three hypotheses are considered to play a role in the etiology of ASH. i) A check-valve mechanism works on the PV that connects a normal scrotal hydrocele to the abdominal cavity and the swollen scrotum extends into the abnormal cavity subsequently [2], [7]. ii) Noncommunicating scrotal hydrocele swells due to increased production or decreased absorption of intra-cystic contents and the hydrocele extends into the abnormal cavity subsequently [7], [8]. iii) A congenital preformed peritoneal diverticulum or defect in the deep inguinal area results in ASH [9]. Ultrasonography can detect ASH if the SBB sign is positive. Additionally, abdominal computed tomography and MRI help differentiate ASH from other diseases such as internal inguinal hernia, hydronephrosis, bladder diverticulum, intrapelvic neuroblastoma, and malignant mesothelioma [10], [11], [12], [13]. In the present case, we diagnosed ASH using MRI and ultrasonography. We reasoned that the ASH was induced by the check-valve mechanism, since laparoscopic findings showed a communication between the ASH and the peritoneum and a clear evidence of PV patency. Many therapeutic interventions have been reported for the treatment of ASH. Generally, open surgical treatment is performed for normal scrotal hydrocele, as very few studies have reported spontaneous remission of a scrotal hydrocele [14]. Additionally, early surgical intervention is recommended to mitigate the damage to the testis [15]. Previously, we made it a rule to perform complete resection of the hydrocele to prevent recurrence. Nevertheless, as with normal scrotal hydroceles, surgical procedures that stop the supply of ascites by high ligation of the PV have recently been widely adopted in patients with ASH. Several surgical approaches such as laparoscopic, inguinal, or scrotal approach have been reported, but an inguinal or scrotal approach may be inadequate to detach the severe adhesion of the spermatic cord and testis in patients with a huge hydrocele. In the present case, we opted to perform early surgical intervention considering the ASH size (8 cm) and adverse effects on testicular development. LPEC helped identify the condition and location of the ASH and allowed safe and reliable operation of the large intrapelvic hydrocele.

LPEC is a helpful and safe surgical technique for patients with ASH. However, there remains a technical problem wherein an internal inguinal ring cannot be confirmed when a swollen hydrocele overhanging the abdominal cavity covers the internal inguinal ring. In such cases, ultrasound-guided puncture from the inguinal region or scrotum to the hydrocele can be used to detect the internal inguinal ring. Nevertheless, if the ultrasound-guided puncture is insufficient, a puncture can be performed laparoscopically using a dissector or a needle. Subsequently, we can perform LPEC following the usual procedure without difficulty. In patients with ASH, severe adhesion is frequently observed between the peritoneum around the internal inguinal ring and the ASH wall. Importantly, LPEC does not require the detachment procedure and can achieve high ligation of the PV by ligating the peritoneum and the ASH wall concomitantly. In ASH patients with no PV patency, a change of approach from LPEC to an open anterior approach should be adopted considering the possibility of hypothesis (ii) described previously.

## 4 Conclusion

This case provides valuable insight into successful LPEC of a large ASH without any complications, highlighting the importance of elucidating the morphological mechanisms and making an accurate diagnosis and the challenges associated with these processes.

## Consent

Written consent was obtained from the patient's parents for writing this case report and accompanying images. Identifying details have been omitted.

## Ethical approval

Ethical approval was not required for this case report in our institution.

## Funding

No funding.

## Guarantor

Dr. Toshifumi Hosoda.

## Research registration number

N/A (this case is not a clinical trial).

## CRediT authorship contribution statement

Dr. Toshifumi Hosoda — corresponding author; collecting the data, writing article, reviewing patient notes, writing articles, analyzing images, and approving final submission.

## Declaration of competing interest

The author declares no financial, personal, or other conflicts of interest that could induce a bias.
